# An NIR-Triggered Au Nanocage Used for Photo-Thermo Therapy of Chronic Wound in Diabetic Rats Through Bacterial Membrane Destruction and Skin Cell Mitochondrial Protection

**DOI:** 10.3389/fphar.2021.779944

**Published:** 2021-11-30

**Authors:** Jiaxin Ding, Binbin Gao, Zhenhua Chen, Xifan Mei

**Affiliations:** ^1^ Jinzhou Medical University, Jinzhou, China; ^2^ Jinzhou Central Hospital, Jinzhou, China

**Keywords:** gold nanocages, epigallocatechin gallate (EGCG), hydrogel, antibacterial, photo-thermotherapy, diabetic wound healing

## Abstract

Bacterial infection and its severe oxidative stress reaction will cause damage to skin cell mitochondria, resulting in long-lasting wound healing and great pain to patients. Thus, delayed wound healing in diabetic patients with *Staphylococcus aureus* infection is a principal challenge worldwide. Therefore, novel biomaterials with multifunction of bacterial membrane destruction and skin cell mitochondrial protection are urgently needed to be developed to address this challenge. In this work, novel gold cage (AuNCs) modified with epigallocatechin gallate (EGCG) were prepared to treat delayed diabetic wounds. The results showed that Au-EGCG had a high and stable photothermal conversion efficiency under near-infrared irradiation, and the scavenging rate of Au-EGCG for *S. aureus* could reach 95%. The production of large amounts of reactive oxygen species (ROS) leads to the disruption of bacterial membranes, inducing bacterial lysis and apoptosis. Meanwhile, Au-EGCG fused into hydrogel (Au-EGCG@H) promoted the migration and proliferation of human umbilical cord endothelial cells, reduced cellular mitochondrial damage and oxidative stress in the presence of infection, and significantly increased the basic fibroblast growth factor expression and vascular endothelial growth factor. In addition, animal studies showed that wound closure was 97.2% after 12 days of treatment, and the healing of chronic diabetic wounds was significantly accelerated. Au-EGCG nanoplatforms were successfully prepared to promote cell migration and angiogenesis in diabetic rats while removing *S. aureus*, reducing oxidative stress in cells, and restoring impaired mitochondrial function. Au-EGCG provides an effective, biocompatible, and multifunctional therapeutic strategy for chronic diabetic wounds.

## Introduction

Long-lasting bacterial infection leads to severe oxidative stress and causes damage to skin cell mitochondria. Thus, antibacterial and mitochondrial protection of skin cells are the key factors for skin wound healing (Li et al., 2007; Wlaschek et al., 2019; Liang et al., 2021). Diabetes can cause damage to the microvascular endothelium and result in tissue hypoxia and ischemia, thus delaying the wound healing and leading to chronic non-healing (YoonYoona et al., 2016; Zhao et al., 2017; Vanaeia et al., 2021). The management of chronic non-healing wounds in diabetes remains a major challenge for doctors, especially since chronic diabetic foot ulcers (DFU) have resulted in more than 73,000 non-traumatic lower-limb amputations, reducing quality of life with huge cost burden (Randeria et al., 2015; Kalan and Brennan, 2018; Arifuzzaman et al., 2019; Chin et al., 2019). Healthy skin is an effective barrier for the protection of the internal organs from pathogens. However, chronic non-healing wounds that are open, moist, and oozing provide the perfect environment for *Staphylococcus aureus* to settle. For the treatment of bacterial infection, the main clinical choice is to use antibiotics, but the excessive use of antibiotics will inevitably lead to drug resistance. Because of the complex healing environment of diabetic skin wounds, it is very promising to develop a multifunctional nanoplatform that can effectively promote wound healing without causing drug resistance.

Photo-thermotherapy (PTT), a new therapeutic strategy, has little effect on the whole body, owing to its penetration ability to the deep tissue and micro-invasiveness, along with a high spatial resolution of the near-infrared laser (NIR). Therefore, PTT can achieve effective local treatment (Qing et al., 2019; Sun et al., 2019; Wang et al., 2019). So far, metal nanostructures (such as nanoparticles, nanorods, and nanolayers) and two-dimensional (2D) nanomaterials (such as black phosphorus, Prussian blue, and copper sulfide) have been used as photothermic agents for near-infrared absorption. These excellent pioneer works disclosed the brilliant prospect of multifunctional nanoplatforms based on PTT strategy (Gupta et al., 2019; Chen et al., 2020a; Tong et al., 2020; Chang et al., 2021). It is well known that gold nanocages are the basic elements used by people for a long time, which is also a kind of photothermic agent. Gold nanocages have unique physicochemical properties (large surface volume ratio, high surface activity, and ultra-small size), are used in combination with two-dimensional nanomaterials to enhance their antibacterial and anti-inflammatory properties, and have been extensively employed in medical and food industries (Hu et al., 2021). In addition, the good biosafety and biocompatibility of gold nanocages have been proven (Wang et al., 2021). Epigallocatechin gallate (EGCG) is a polyphenol bioactive compound that has an inhibitory effect on the growth of Gram-negative bacteria and Gram-positive bacteria. EGCG also has the biological functions of anti-oxidation, enhancing immunity, and effectively promoting wound healing. However, the application of green tea polyphenols (GTP) is limited by its weak lipo-solubility, easy oxidation, and low activity in an alkaline solution (Krook and Hagerman, 2012; Li et al., 2012; Barbalho et al., 2019). Hydrogels are macromolecular biomaterials that absorb large amounts of water or other water-soluble substances; have excellent biocompatibility and biodegradability; and can deliver functional drugs, molecules, and cells in a variety of therapies. Hydrogel has been widely applied in the field of tissue engineering and medicine as the carrier of the sustained-release drug to protect the injured part from harmful stimulation, to keep the injured part moist, and to improve the drug utilization rate (Zhao et al., 2019a; Wu et al., 2019; Zhang et al., 2019; Chen et al., 2020b).

**SCHEME 1:**
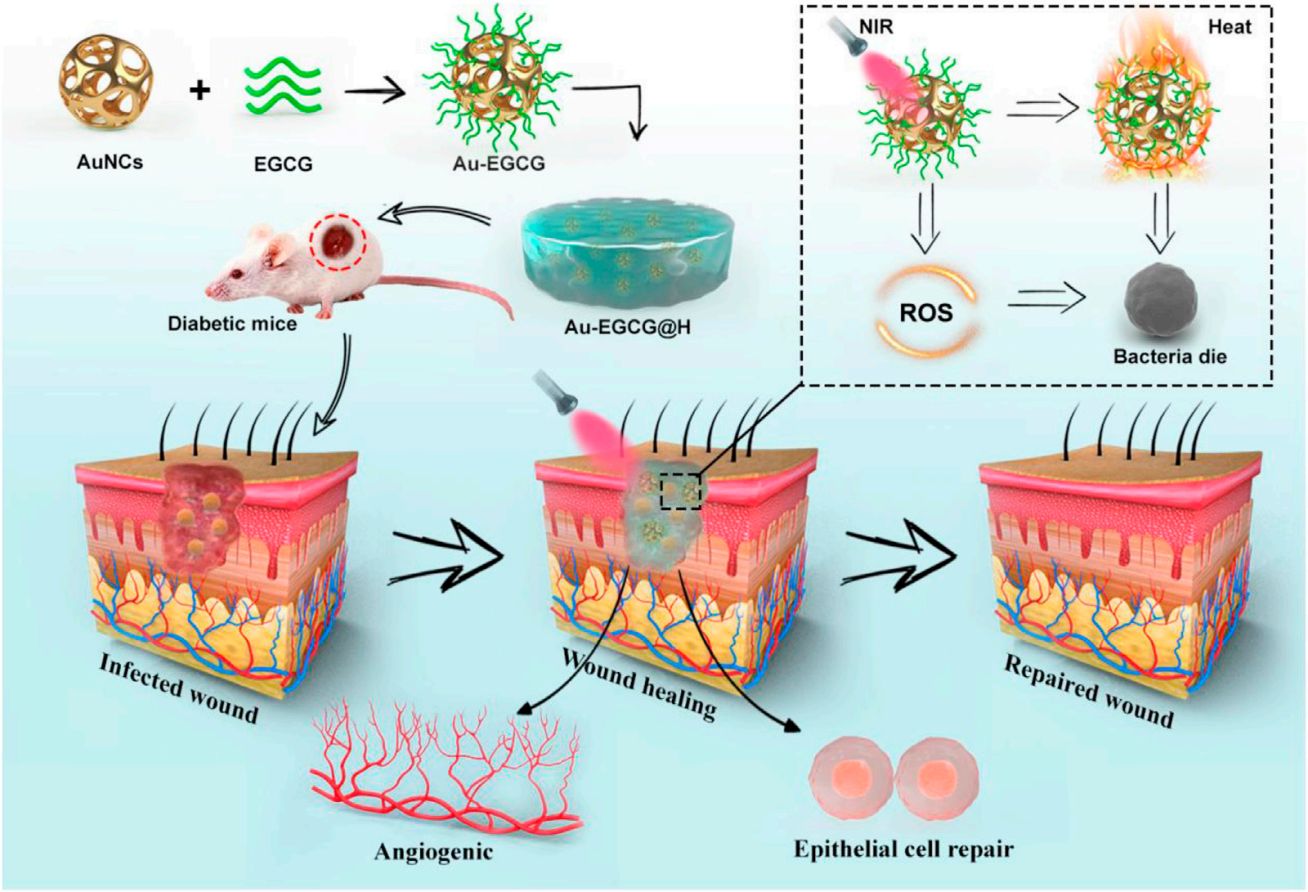
The schematic illustration of the synthesis of Au-EGCG@H nanocomposite and the process of sterilization and stimulation of cell behaviors under NIR irradiation that can facilitate the regeneration of skin cells, involve in the regeneration of skin actively to promote the healing of chronic wounds of infected bacteria.

A NIR-triggered Au nanocage with functions of bacterial membrane destruction and skin cell mitochondrial protection was developed to solve these problems, as shown in Scheme 1. A stable drug dispersion system was prepared by loading EGCG onto hollow porous gold cage (AuNCs) and coating it uniformly in a novel hydrogel with excellent biocompatibility. We use Au-EGCG fused into hydrogel (Au-EGCG@H) in diabetic wounds with *Staphylococcus aureus* infection. The results showed that Au-EGCG@H possessed a high and stable photothermal conversion efficiency under NIR irradiation, and the generation of a large amount of reactive oxygen species (ROS) led to the destruction of the bacterial membranes, thereby inducing bacterial lysis and apoptosis. Meanwhile, Au-EGCG@H promoted the migration and proliferation of human umbilical cord endothelial cells, reduced oxidative stress in cells under infection, restored cellular mitochondrial function, and significantly increased the expression of basic fibroblast growth factor and vascular endothelial growth factor. The ability to promote epithelial repair will then be further tested in a combination of cell and animal studies. We hope that this composite nanomaterial system can protect the chronic non-healing wounds of diabetes from the interference of the external environment and at the same time it can promote the proliferation of endothelial cells and the repair of epithelia by eliminating bacteria through photothermal therapy, accelerating the repair of chronic non-healing wounds. More importantly, we also hope to develop a new, economical, safe, and multifunctional combination as an effective nanodrug platform treatment strategy.

## Materials and Methods

### Materials

The fetal bovine serum (FBS), 3-(4,5-dimethylthiazol-2-yl)-2,5-diphenyl tetrazolium bromide (MTT), and Dulbecco’s modified Eagle’s medium (DMEM) were provided by Gibco (United States). From Beyotime, we obtained 2,7-dichlorodihydrofluorescein diacetate (DCFH-DA). The primary antibodies for glyceraldehyde 3-phosphate dehydrogenase (GAPDH), vascular endothelial growth factor (VEGF), and basic fibroblast growth factor (bFGF) were offered by Cell Signaling Technology (United States). From the American Type Culture Collection (ATCC), the human umbilical vein endothelial cells (HUVECs) and human skin keratinocyte cells (HACT) can be acquired. The ultrapure water was utilized in the whole experiment, which was acquired from the system of Milli-Q. The expression of relevant proteins in the cells was observed by confocal laser scanning microscopy (CLSM; Leica TSC SP5 confocal unit).

### Preparation of Au-EGCG@H

We prepared the AuNCs and then combined them with EGCG to form Au-EGCG fused into the hydrogel. In short, we put 10 ml PVP (4 mg/ml) into a three-port flask and added 4 ml of silver nanoparticles. Then, the solution was heated in an oil bath (100°C, 500 r/min). After 15 min, HAuCl_4_ (0.0543 mg/ml) was subsequently slowly added at 3 ml/min until the color of the reaction was stable. Washing the samples with saturated NaCl solution and deionized water, respectively, was done after cooling to room temperature. Subsequently, EGCG (2 ml, 50 μg/ml) was added to the prepared Au solution and then mixed for 2 h at room temperature (500 r/min) to form Au-EGCG. The samples were collected by centrifugation (12,000 rmp, 20 min). The hydrogel was prepared by adding 0.1 g to 10 ml PVA solution (8%), and then, the solution was stirred for 1 h (50°C, 350 r/min). Finally, Au-EGCG were fused into the hydrogel to obtain Au-EGCG@H.

### Characterization

Dynamic laser scattering (DLS, Malvern, NanoZS90, Worcestershire, United Kingdom) and transmission electron microscopy (TEM, JEM-1200EX, Tokyo, Japan) were respectively employed to characterize the size and morphology of AuNCs. The crystallographic structure of AuNCs was demonstrated using a PXRD analysis (Shimadzu, Kyoto, Japan) applying Cu K radiation. Fourier transform infrared spectroscopy (FTIR, Shimadzu, Kyoto, Japan) was employed to explore the component of Au-EGCG@H utilizing the KBr disk approach. The fluorescence photometer (F97PRO, Shanghai, China) and UV-vis spectrophotometer (PerkinElmer Lambda 605S UV-vis spectrometer) were exploited to acquire fluorescence data and ultraviolet-visible data.

### Photothermal Performance and Photothermal Stability of Au-EGCG@H

Thermo photographic research was used to assess the photothermal properties of Au-EGCG@H. We added Au-EGCG@H at various concentrations (50, 100, or 200 μg/ml), AuNCs, and water into centrifuge tubes. Then, we irradiated them with NIR laser (1 W/cm^2^, 808 nm) for 10 min while recording the thermal images and temperature with an NIR thermal imager (ABFRX500) every 30 s. To evaluate the photothermal stability of Au-EGCG@H, AuNCs and Au-EGCG@H were warmed and cooled for three cycles. Each cycle irradiated them for 10 min while recording the temperature at intervals of 30 s.

### Scratch Assay

The migration capability of HUVECs was measured *via* scratch detection. HUVECs were inoculated into 24-well plates (1 × 10^6^ cells/well) and starved with FBS-free medium at 37°C (5% CO_2_, 95% humidity). The tip of a sterile pipette was used, and the cells were scratched with a line at the bottom of the plate. Phosphate-buffered saline (PBS) was used to wash the floating debris and cells. Subsequently, HUVECs were treated with Au-EGCG@H, Au@H, or PBS, and then, they were incubated under a temperature of 37°C. Afterward, 4% paraformaldehyde (PFA) was utilized to fix HUVECs, and then, HUVECs were treated with 0.1% Triton X-100 (Sigma-Aldrich, United States) and stained by DAPI. An inverted Leica fluorescence microscope was exploited to take the migration images. After determining the distance of scratch healing, the migration images were quantified.

### Immunofluorescence Staining

In each group, after the incubation, the HUVECs were cleaned three times by utilizing PBS. Afterward, the HUVECs were fixed for half an hour in 4% PFA. Next, the cells were cleaned in PBS for three times and incubated using Triton X-100 for 20 min. The cells were subsequently incubated utilizing goat serum for 2 h. After that, the treatment of the cells was conducted *via* the bFGF antibodies and primary anti-VEGF antibodies at 4°C overnight. Next, the incubation of cells was performed through Alexa Fluor 488 goat anti-rabbit IgG or Alexa Fluor 594 goat anti-mouse IgG at RT for 2 h. In the end, DAPI (Invitrogen, United States) was utilized to stain the nuclei for 20 min.

### Au-EGCG@H *In Vitro* Antibacterial Efficiency

The antibacterial ability of near-infrared laser irradiation combined with nanomaterials was evaluated with *S. aureus*. In brief, 200 μl of Au-EGCG@H was mixed with 800 μl bacterial solution and incubated for 30 min. Afterward, we irradiated the solution by applying the NIR laser (808 nm, 2.5 W/cm^2^, 5 min). Next, the treated bacterial suspension was diluted and then diffused onto the nutrient agars. After incubation for 1 day at 37°C, we can count the number of cloned bacteria.

### Detection of ROS

The fluorescent probe DCFH-DA was provided by Beyotime to detect the intracellular levels of ROS of treated bacteria. In brief, *S. aureus* cells were treated with PBS, Au-EGCG@H, or Au-EGCG@H by the irradiation of NIR (5 min, 2.5 W/cm^2^) at 37°C for 30 min. Then, in various treatments, *S. aureus* cells were incubated in darkness for 20 min through the probe of DCFH-DA. The samples were ultimately visualized *via* an inverted Leica fluorescence microscope to obtain bacterial images.

### *In Vivo* Treatment of Diabetic Rat *S. aureus*-Infected Wounds

We acquired the female SD rats from Jinzhou Medical University Animal Center. All the studies were conducted using female SD rats with weight between 200 and 220 g. The diabetes animal model was constructed with streptozocin (80 mg/kg, STZ; Sigma, Louis, MO, United States). After anesthesia and disinfection, the dorsal area was fully shaved, and then, a 1.5-cm full-thickness excision wound was formed on the back. *S. aureus* was selected as a bacterial strain for infection. Afterward, *S. aureus* suspension (400 μl) was added to the circular wound to infect all of the rats. All of the animal researches were performed adhering to the Guidelines for Care and Use of Laboratory Animals of Jinzhou Medical University, and the researches were authorized through the Animal Ethics Committee of Jinzhou Medical University.

### Western Blot Analysis

The skin tissues around the wound were harvested on the 8th day after the injury and then dissolved by using the RIPA buffer (Beyotime, China). The supernatant was subsequently harvested through centrifugation at 12,000 rpm, at 4°C for 15 min, and the concentration of protein was determined *via* the BCA Protein Assay Kit (Solarbio, China). Each channel is loaded with an equal amount of protein lysate isolated on an acrylamide gel (10%) and subsequently transferred to the membrane of PVDF. After blocking, corresponding primary antibodies, including VEGF, bFGF, and GAPDH, were incubated at 4°C for 12 h. The GAPDH expression was exploited as a control. Images were captured by Alpha Innotech Photo-documentation System.

### Tissue Histology

The sections of skin tissue were taken on the 12th day after injury. The skin tissue was soaked in PFA (4%) for 2 days, dehydrated in graded alcohol, and finally degreased in xylene. After embedding the samples in paraffin, the samples were cut into sections (4 µm), and they were subsequently stained by hematoxylin and eosin (H&E). All images were captured using a light microscope.

### Statistical Analysis

All of the studies were implemented in triplicate. The Origin 9.0 software was utilized to plot all of the graphs. Multiple comparisons were conducted with one-way ANOVA. A *P* value less than 0.05 was regarded statistically significant.

## Result and Discussion

### Preparation and Characterization of Au-EGCG@H

**FIGURE 1:**
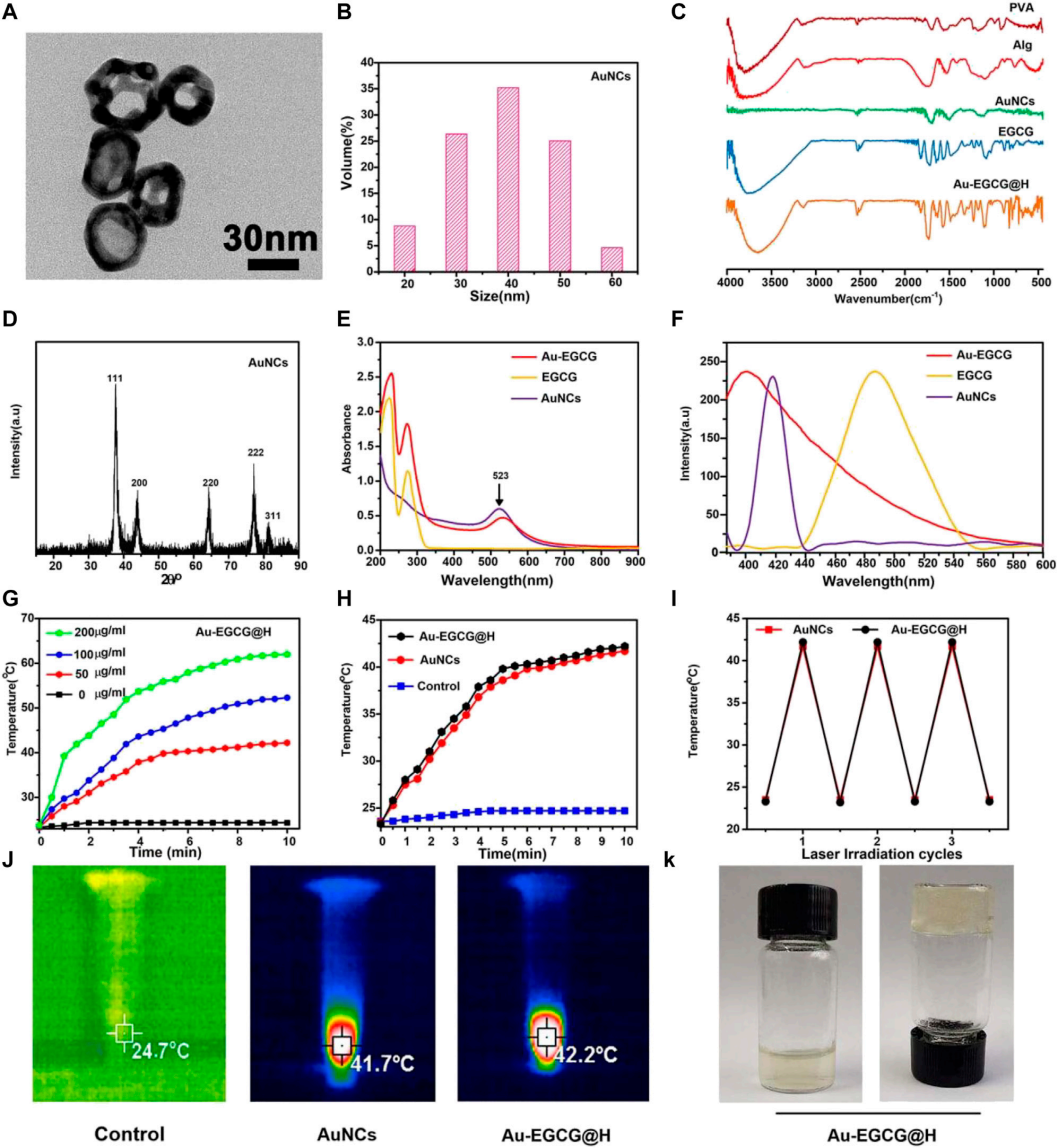
TEM images **(A)**; DLS size distribution of AuNCs **(B)**; Fourier Transform Infra-Red spectra **(C)**; the patterns of XRD **(D)**; Ultraviolet-visible **(E)**; Steady-state fluorescence spectra of the samples **(F)**; Heating curves of Au-EGCG@H at various concentrations **(G)**; Heating curves of Au-EGCG@H, AuNCs, and water **(H)**; Photostability of Au-EGCG@H and AuNCs **(I)**; Thermal images of Au-EGCG@H, AuNCs, and water exposed to laser (1 W/cm^2^, 808 nm) for 10 min **(J)**; Digital photos of Au-EGCG@H **(K)**.

DLS and TEM were respectively employed to characterize the size distribution and morphology of the created AuNCs. The TEM image (Figure 1A) showed that AuNCs possess excellent uniformity, regular morphology, and a hollow and porous (8 nm) structure. DLS (Figure 1B) proved that the AuNCs’ diameter was about 40 nm, and their polydispersity index (PDI) was <0.1, further demonstrating that these AuNCs are uniform. Of the AuNCs, the particle size range of 40 ± 10.5 nm accounts for about 80%. The FTIR spectrum confirmed the successful formation of Au-EGCG@H. As reported in Figure 1C, compared with the FTIR of AuNCs, the strong and wide absorption peaks of Au-EGCG@H at 3,755 cm^−1^ was owing to the O–H stretching vibration on aromatic ring, and the wide absorption band between 1,600 and 1,450 cm^−1^ was associated with the stretching vibration C=C in EGCG benzene ring; the absorption peaks at 1,327 cm^−1^ were also the basic characteristics of EGCG, that is, C–O stretching vibration, and the role of hydrogen bond, indicating that Au-EGCG has been successfully prepared and fused into the hydrogel. The successful preparation of gold nanocages means that the hydrogel system is photo-responsive.

The XRD patterns (Figure 1D) of AuNCs had sharp diffraction peaks at 38.2°, 44.97°, 64.67°, and 77.64°, which correspond to (111), (200), (220), and (311) crystal surfaces (JCPDS 4–0784), suggesting the crystalline nature of the AuNCs. The UV-vis spectroscopy demonstrated the formation of Au-EGCG. As presented in Figure 1E, the surface plasmon resonance absorption of AuNCs at 523 nm and the characteristic absorption peak of EGCG at 273 nm were found in the Au-EGCG ultraviolet-visible spectrum, indicating successfully prepared Au-EGCG. Fluorescence spectra further indicated that EGCG and AuNCs synthesized Au-EGCG (Figure 1F). The fluorescence spectrum of AuNCs appeared at a peak at 418 nm, and the fluorescence spectrum of EGCG displayed a peak at 486 nm. The emission peak of Au-EGCG (400 nm) revealed a diverse blue shift. These outcomes demonstrated the successful preparation of Au-EGCG. To estimate the photothermal performance of Au-EGCG@H, we measured the photothermal heating curves of Au-EGCG@H during 808-nm laser irradiation (1 W/cm^2^). As can be seen in Figure 1G, Au-EGCG@H showed a concentration-dependent photothermal effect. The highest temperature increased significantly as the concentration of Au-EGCG@H increased at the same irradiation condition. As indicated in Figure 1H, in contrast to the control group, the maximum temperature of AuNCs and Au-EGCG@H upregulated evidently, as the increasing time of irradiation and the maximum temperature of Au-EGCG@H are slightly higher than those of AuNCs at an identical irradiation condition. These outcomes suggested that Au-EGCG fused into the hydrogel does not influence the AuNC photothermal property. To estimate the photothermal conversion stability of Au-EGCG@H, the heat production efficiency of AuNCs and Au-EGCG@H was assessed after three cycles of heating and cooling processes. As detected (Figure 1I), after three cycles, the temperature increase of AuNCs and Au-EGCG@H did not change significantly, reflecting the excellent photothermal stability of Au-EGCG@H. In Figure 1J, the highest temperature of AuNCs and Au-EGCG@H could reach 41.7°C and 42.2°C, respectively. These results demonstrated that Au-EGCG@H had excellent photothermal performance, which is beneficial for the photothermal elimination of bacteria. According to Figure 1K, gelation was successfully obtained after adding Au-EGCG to the hydrogel, which indicates that the prepared Au-EGCG@H has good gelation properties. This indicates that we have successfully prepared a solid hydrogel, which facilitates fixation to the wound after application to the skin surface.

### *In Vitro* Antibacterial Properties of Au-EGCG@H

**FIGURE 2:**
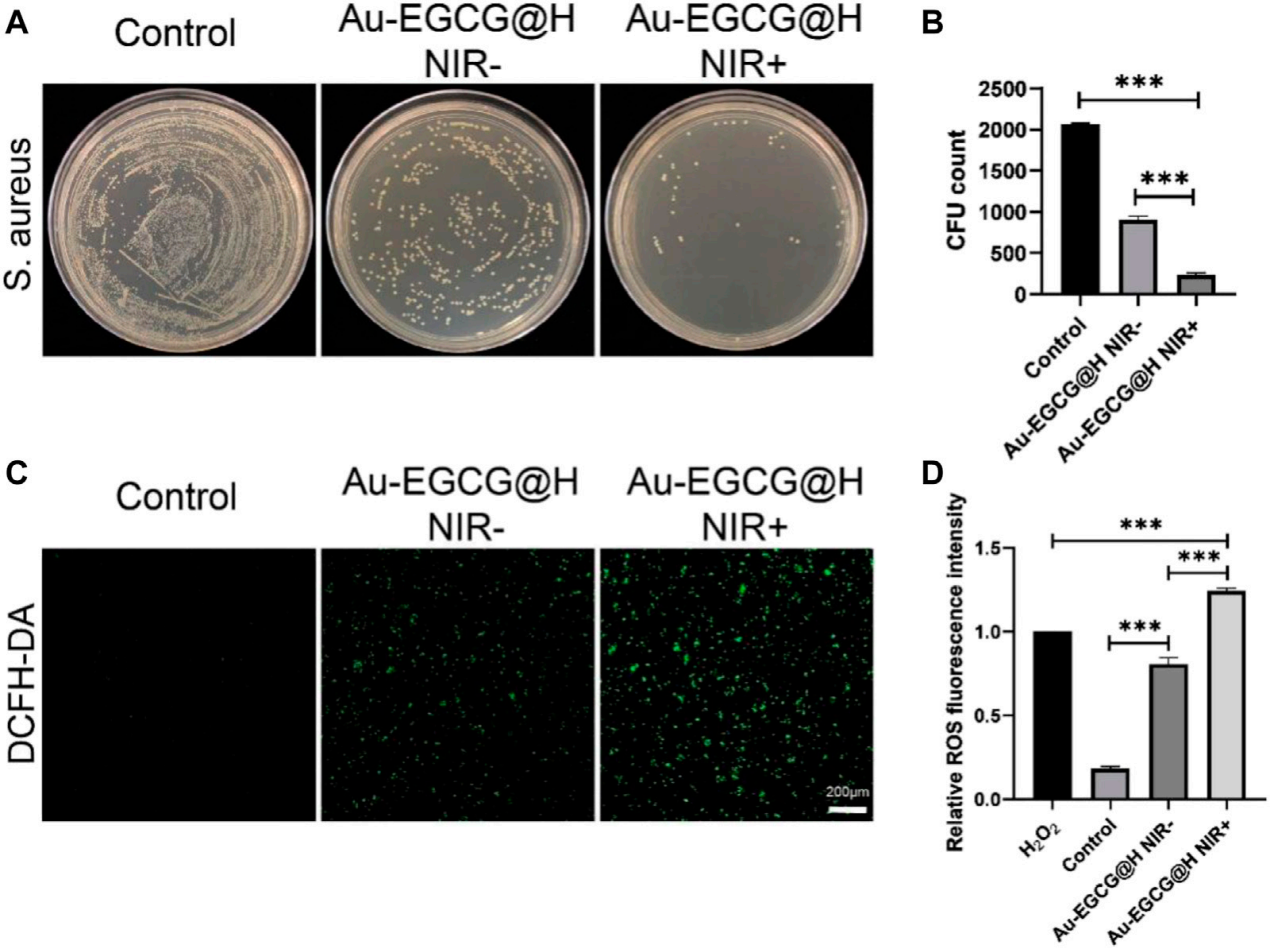
effectiveness of Au-EGCG@H laser irradiation for sterilization. **(A)** *S. aureus* agar plate photos of distinct treatments. **(B)** The corresponding colony-forming unit (CFU) amount of *S. aureus* with different treatments. **(C)** *In vitro* reactive oxygen species (ROS) effects with various treatments. Stained by 2,7-dichlorodihydrofluorescein diacetate (DCFH-DA), bar = 20 μm. **(D)** The relevant statistical histogram revealing the relative ROS fluorescence intensity. *p < 0.05, **p < 0.01, ***p < 0.001.

The antibacterial activity of Au-EGCG@H was investigated through the spread plate method. As we expected, treatment with PBS had no distinct effect on the bacterial viability (Figure 2A). The viability of bacteria showed an obvious decrease upon being treated with Au-EGCG@H, while it was more significant when NIR was exerted (Figure 2B). This means that Au-EGCG@H with NIR could more effectively inhibit bacterial survival and suppress the growth of *S. aureus*. ROS can oxidize and modify nucleic acid, protein, lipid, and other cell components, leading to genomic damage, enzyme dysfunction, membrane fluidity changes, and ultimately bacterial death (Panda et al., 2018; Wang et al., 2017; Lee et al., 2014). To investigate the level of ROS after irradiation, we can quantify the level of ROS *via* the measurement of the 2,7-dichlorofluorescein (DCF) fluorescence intensity. We found that after treatment with PBS, almost no green fluorescent spots were observed in the bacteria, indicating no induction of ROS. ROS generation was present in the Au-EGCG@H group, while NIR irradiation significantly enhanced the production of ROS level after treatment (Figure 2C, D). Various theories have been put forward about how AuNCs work against bacteria. We believe that AuNCs can interact with bacterial cell walls and penetrate them, resulting in structural damage, cellular destruction, and bacterial death (Burdușel et al., 2018). ROS induction is thought to be another mechanism by which AuNCs induce bacterial death. At the same time, the phenolic hydroxyl group of EGCG can bind to the bacterial lipid bilayer and the amino and carboxyl groups in the bacterial membrane protein, thus destroying the integrity of the bacterial membrane (Steinmann et al., 2013; Xiong et al., 2017). We believe this combined effect contributes to effective antimicrobial therapy. In addition to this, we stimulated HUVEC with LPS to simulate bacterial infections *in vivo*. The intracellular ROS was detected by a fluorescence microplate reader, and the results showed that the intracellular ROS content was significantly reduced in the Au-EGCG-treated group (Supplementary Figure S1).

### The Effect of Au-EGCG@H on Cell Migration and Proliferation *In Vitro*

**FIGURE 3:**
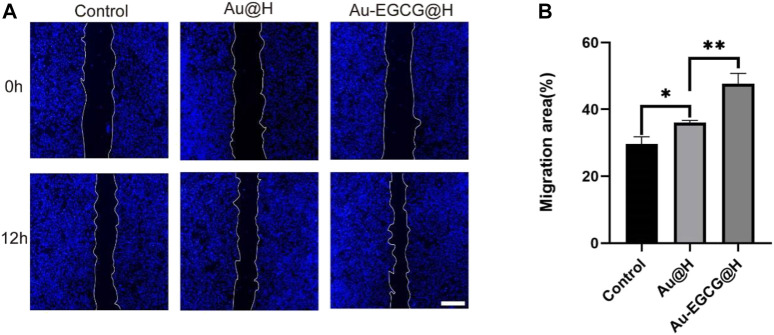
The human umbilical vein endothelial cell (HUVEC) migration treated by Au-EGCG@H and Au@H, respectively. **(A)** The scratch-wound assay in different treatments, bar = 200 μm. **(B)** Migration area analysis. *p < 0.05, **p < 0.01, ***p < 0.001.

Subsequently, we assayed the toxicity of AuNCs as well as Au-EGCG on HUVECs by MTT assay. As shown in Supplementary Figure S1, cell viability decreased slightly with increasing concentrations, while at the highest concentration of AuNCs (50 μm), it decreased to 88%, indicating lower toxicity. Notably, the cytotoxicity of Au-EGCG was lower than that of AuNCs when the concentrations were the same (Supplementary Figure S2). Next, to demonstrate the role of Au-EGCG@H in accelerating skin tissue repair, we selected HUVEC for scratch test *in vitro*. The degree of migration showed significant differences after different treatments of cell scratch (Figure 3A). The cells exhibited the strongest migration after treatment with Au-EGCG@H. The results of the quantitative analysis were also consistent with the migration observations, with the Au-EGCG@H group showing the highest migration rate (Figure 3B).

**FIGURE 4:**
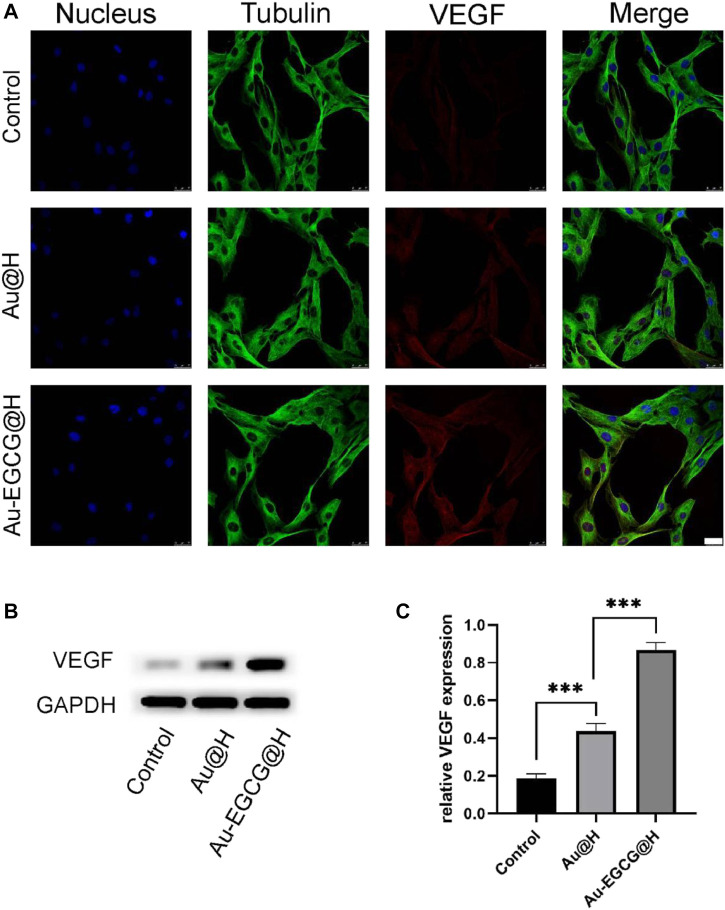
The expression analysis of vascular endothelial growth factor (VEGF) under different treatments. **(A)** The images of confocal laser scanning microscopy (CLSM) for the VEGF expression analysis, bar = 25 μm. **(B)** The VEGF protein expression level was explored through western blot. **(C)** Quantification analysis of the VEGF protein. *p < 0.05, **p < 0.01, ***p < 0.001.

In addition, CLSM was employed to observe the expression of the relevant proteins in the cells. Our result showed that Au-EGCG further enhanced the expression of VEGF in HUVECs (Figure 4A). To further confirm our speculation, we next examined protein levels of VEGF in the wound region by utilizing western blot (Figure 4B). By the CLSM observations, the VEGF protein expression in the two groups treated with Au-EGCG@H and Au@H, respectively, was higher than the expression of the control group, and the VEGF expression in the Au-EGCG@H-treated group was higher than the expression in the Au@H-treated group (Figure 4C).

**FIGURE 5:**
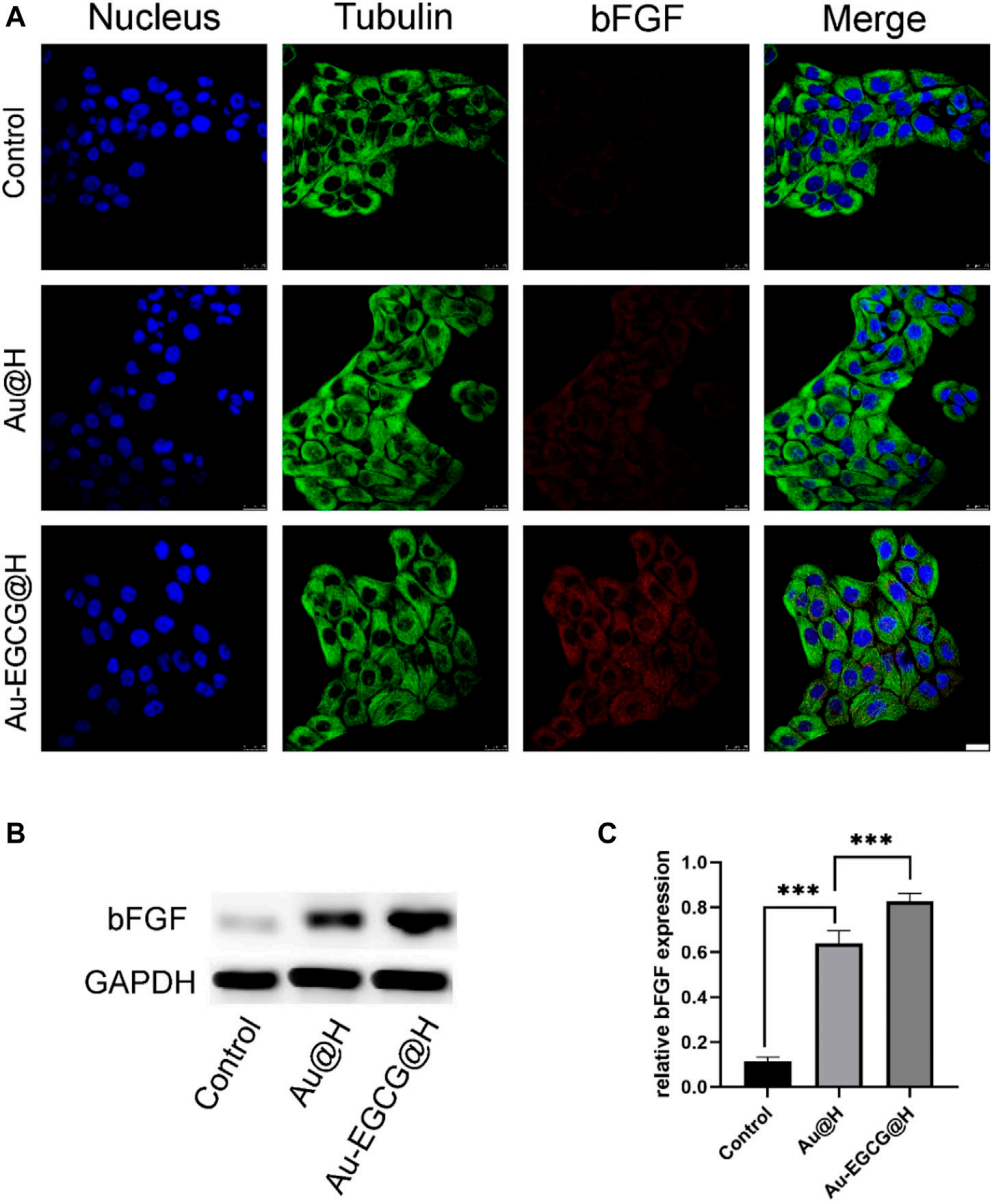
The expression analysis of basic fibroblast growth factor (bFGF) under different treatments. **(A)** The images of confocal laser scanning microscopy (CLSM) for the bFGF expression analysis, bar = 25 μm. **(B)** The expression level of bFGF protein was analyzed by western blot. **(C)** Quantification analysis of the bFGF protein. *p < 0.05, **p < 0.01, ***p < 0.001.

Considering that epithelial cells are an integral part of repairing skin defects, we used CLSM to examine bFGF expression in HACAT cells. The growth factor of bFGF stimulates cell proliferation and migration, thereby promoting wound healing and tissue repair. According to the fluorescence results (Figure 5A), bFGF in the AU-EGCG@H group showed a trend towards increased relative protein expression in comparison with the control group. Thereafter, we examined and quantified the expression of the relevant proteins by western blot (Figure 5B). After different biological treatments, the amount of bFGF protein increased to different degrees in Au@H- and Au-EGCG@H-treated tissues (Figure 5C). The highest expression levels were found in the Au-EGCG@H group (Figure 5C). This demonstrates that the Au-EGCG@H has a higher growth-promoting ability than Au@H alone.

In the process of wound healing, angiogenesis, the proliferation of keratinocyte, and the migration of endothelial cells are critical for the generation (re-epithelialization) of the novel epidermal layers and the restoration of tissue integrity (Chen et al., 2018). EGCG has also been shown to be an excellent mediator of cell proliferation in accelerating wound healing. Simultaneously, EGCG has beneficial pharmacological effects as anti-bacterial, anti-inflammatory, and angiogenesis promoter. Our functional tests *in vitro* suggested that AU-EGCG@H enhanced the migration and proliferation of HUVECS. Meanwhile, we determined an increase in a related vasculogenic protein expression, an early marker of angiogenesis.

### Evaluation of Materials for Promoting Wound Healing in Diabetic Rats Infected With *S. aureus*

**FIGURE 6:**
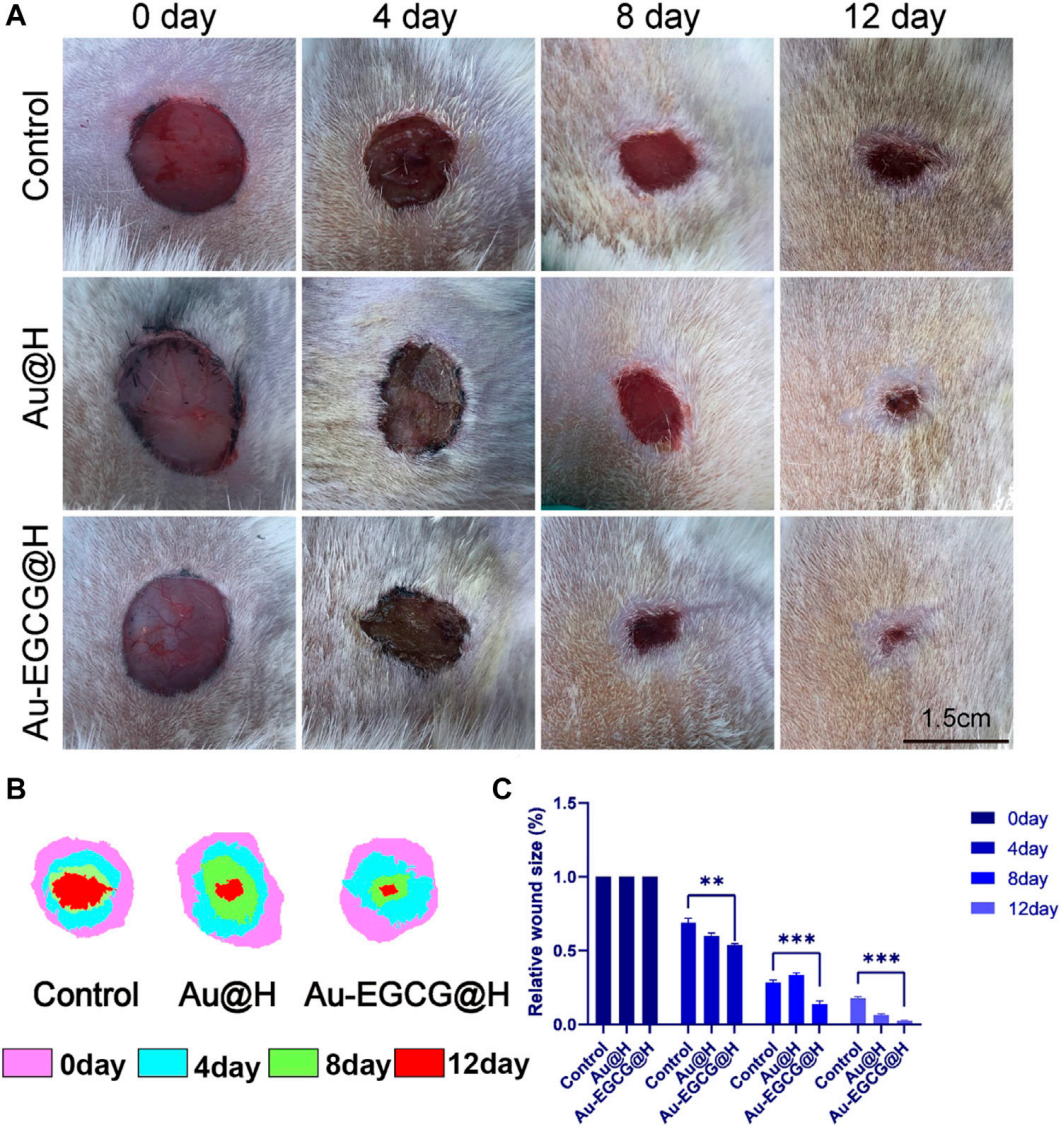
Gross changes of the wound site. **(A)** The image of the diabetic skin wound after operation, bar = 1.5 cm. **(B)** The wound-bed closure traces within 12 days of each treatment. **(C)** Wound closure rate analysis. *p < 0.05, **p < 0.01, ***p < 0.001.

In order to investigate the diabetic wound-healing process, a full-length wound is formed on the back of the rat as described in the *Materials and Methods*. Different groups were treated with Au-EGCG@H, Au@H, and vehicle (control), and wound closure sizes were photographed on postoperative days 0, 4, 8, and 12 (Figure 6A). TPN@H revealed significant influence against wound healing in rats with diabetes (Figure 6B). It is worth mentioning that Au-EGCG@H significantly promoted wound healing in diabetic rats (Figure 6C). Images of the wound area exhibited a remarkable reduction in the size of wound closure over time in all groups, particularly on postoperative days 8 and 12. Also, the wound size was down-regulated in the group of Au-EGCG@H, in contrast to the control group, indicating that Au-EGCG@H accelerates wound healing.

**FIGURE 7:**
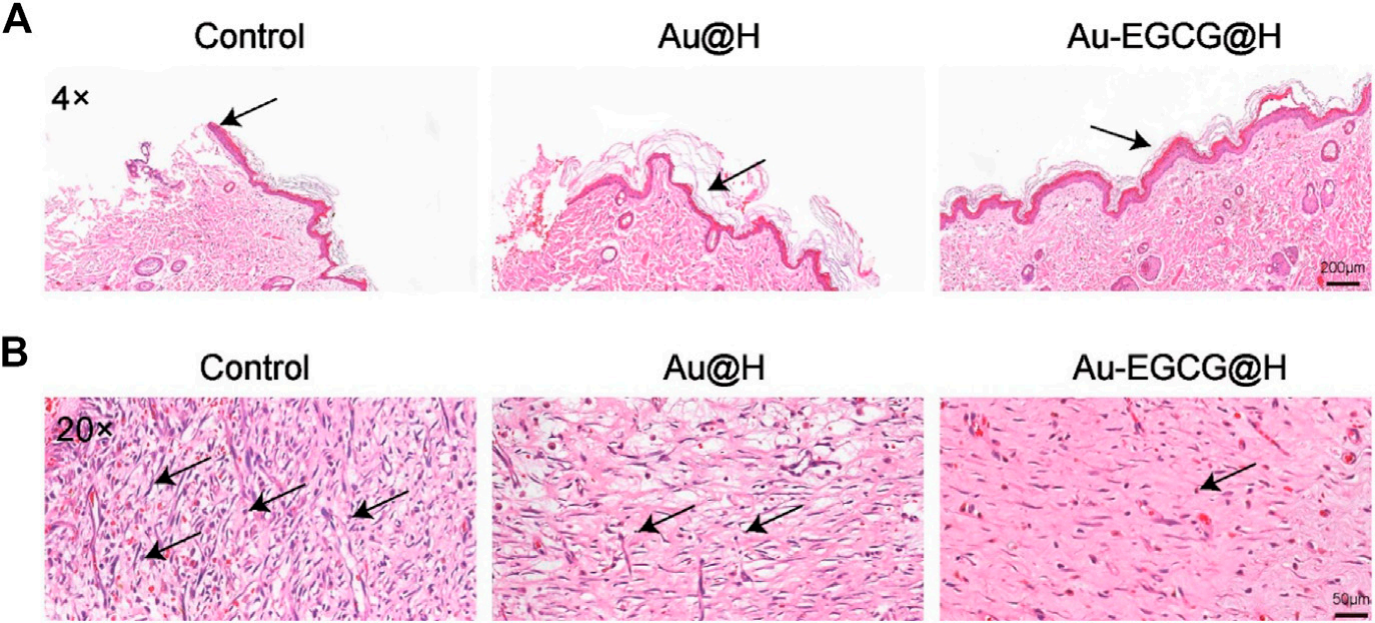
Histological changes at the wound site. **(A)** The staining of hematoxylin and eosin (H&E) in wound sites after 12 days of distinct treatments, bar = 200 μm. **(B)** Local enlargement of H&E stain, bar = 50 μm.

Inflammatory response possesses a significant effect in the repair together with the regeneration of chronic healing wounds in diabetic patients with bacterial infection. Inflammatory reaction also operates repair mechanisms that promote epithelial cell proliferation. However, excessive inflammation could mitigate wound healing and disrupt the normal wound-healing sequence (Zhao et al., 2019b). To evaluate the histological changes in the wounds, we utilized H&E staining to observe the healing skin microstructure. In comparison with the group of Au-EGCG@H, the control group did not generate complete epithelial tissue. The group of Au@H already had re-epithelialization, but the epithelium in the group of Au-EGCG@H was more regular and smoother than that in the Au@H group (Figure 7A, the arrow points to the repaired epithelium). In addition, we examined wound healing in rats by Masson’s staining (Supplementary Figure S3). The results showed that Au-EGCG@H had an excellent promotion effect on wound healing in rats. Subsequent H&E staining inflammatory analysis indicates there are many inflammatory cells in the control group, while the inflammatory cell number in wounds of Au-EGCG@H and consolidation treatment Au@H decreased significantly (Figure 7B, the arrow points to infiltrating inflammatory cells). The most significant reduction in inflammatory cells was seen in the group treated with Au-EGCG@H. All of these outcomes confirmed the effectiveness of Au-EGCG@H photothermal treatment mediated by NIR irradiation to enhance epidermis regeneration and anti-inflammatory properties by reducing the burden of bacterial infection.

## Conclusion

In conclusion, a NIR-triggered Au nanocage with functions of bacterial membrane destruction and skin cell mitochondrial protection was developed in this work. The designed nanohydrogel effectively slowed down the oxidation of EGCG and prolonged its action time. Under the irradiation of 808-nm laser, Au-EGCG@H has good photo-thermal stability, and the photo-thermal properties are improved obviously. EGCG and AuNCs synergistically promoted wound angiogenesis, reduced oxidative stress generated within vascular endothelial cells, and preserved impaired mitochondria. After 12 days of treatment, the diabetic rats in the Au-EGCG@H group had excellent wound-healing results. This photo-responsive, spreadable antimicrobial hydrogel system has a certain degree of adhesion to the wound, which is more prominent than ordinary hydrogels. In addition to this, the composite material with antimicrobial ability has a better repair effect compared to regular dressings. Thus, the AU-EGCG@H nanocomposite offers a promising strategy for efficient wound healing in diabetes.

## Data Availability

The original contributions presented in the study are included in the article/Supplementary Material, further inquiries can be directed to the corresponding authors.
