# Supplementary material for: An NIR-Triggered Au Nanocage Used for Photo-Thermo Therapy of Chronic Wound in Diabetic Rats Through Bacterial Membrane Destruction and Skin Cell Mitochondrial Protection
Source: Front Pharmacol. 2021 Nov 30;12:779944. doi: 10.3389/fphar.2021.779944 (PMC8671044; doi:10.3389/fphar.2021.779944)
Supplement: Supplementary file 1 [file DataSheet1.PDF]

Supporting Information

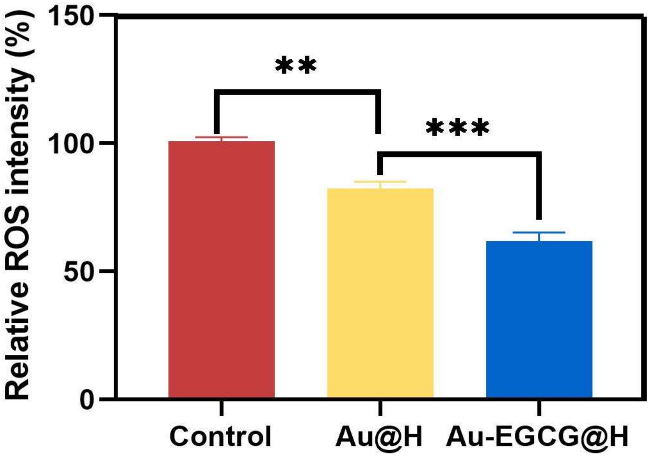

Fig. S1. Evaluation of the extent of intracellular ROS.

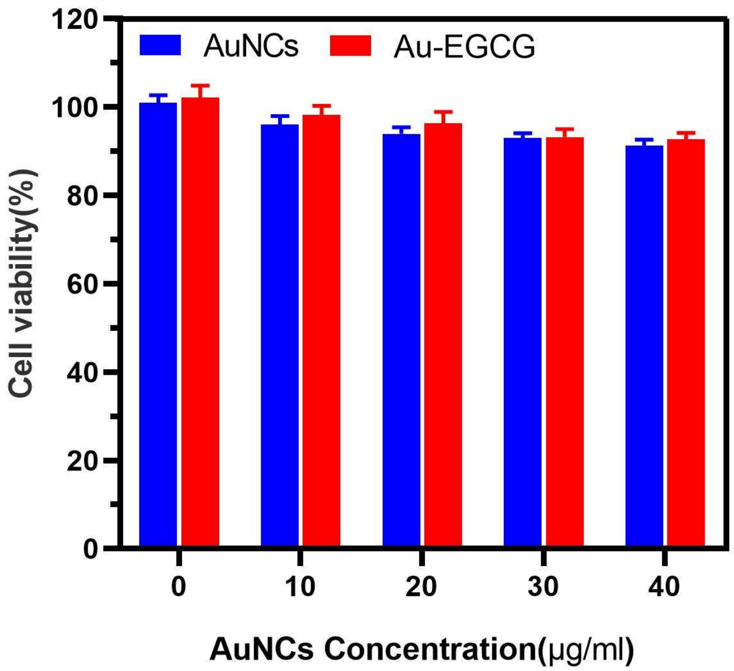

Fig. S2. Cell viability of HUVEC treated with AuNCs and Au-EGCG.

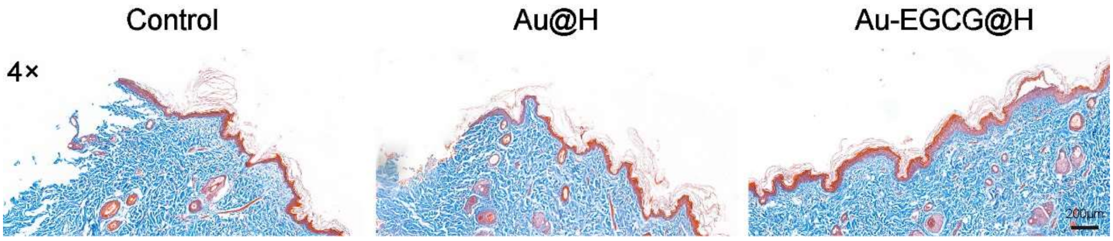

Fig. S3. Masson staining of the wound tissues.
